# Tamm Plasmon Polariton Biosensors Based on Porous Silicon: Design, Validation and Analysis

**DOI:** 10.3390/bios13121026

**Published:** 2023-12-11

**Authors:** Guoguang Rong, Mohamad Sawan

**Affiliations:** CenBRAIN Neurotech Center of Excellence, School of Engineering, Westlake University, 600 Dunyu Road, Xihu District, Hangzhou 310030, China; rongguoguang@westlake.edu.cn

**Keywords:** Tamm Plasmon Polariton, optical biosensor, porous silicon, nucleocapsid protein, SARS-CoV-2

## Abstract

Tamm Plasmon Polariton (TPP) is a nanophotonic phenomenon that has attracted much attention due to its spatial strong field confinement, ease of mode excitation, and polarization independence. TPP has applications in sensing, storage, lasing, perfect absorber, solar cell, nonlinear optics, and many others. In this work, we demonstrate a biosensing platform based on TPP resonant mode. Both theoretical analyses based on the transfer matrix method and experimental validation through nonspecific detection of liquids of different refractive indices and specific detection of SARS-CoV-2 nucleocapsid protein (N-protein) are presented. Results show that the TPP biosensor has high sensitivity and good specificity. For N-protein detection, the sensitivity can be up to 1.5 nm/(µg/mL), and the limit of detection can reach down to 7 ng/mL with a spectrometer of 0.01 nm resolution in wavelength shift. Both nonspecific detection of R.I. liquids and specific detection of N-protein have been simulated and compared with experimental results to demonstrate consistency. This work paves the way for design, optimization, fabrication, characterization, and performance analysis of TPP based biosensors.

## 1. Introduction

Biosensors have wide applications in biomedicine [1], food safety [2], environment protection [3], and biosafety [4]. Optical biosensors have the advantages of high sensitivity, good specificity, fast response, and low cost [5]. Researchers have proposed optical biosensors based on optic fiber [6], optical waveguide [7], ring resonator [8], optical interferometer [9], photonic crystal [10], surface plasmon resonance (SPR) [11], and localized surface plasmon resonance (LSPR) [12]. Optical biosensors have been demonstrated for the detection of bacteria [13], virus [14], DNA [15], proteins [16], and small molecules [17].

Tamm Plasmon Polariton (TPP) is an electromagnetic surface state confined at the interface between plasmonic material and one-dimensional photonic crystal or Distributed Bragg Reflector (DBR) [18]. It can be excited by incident light in air without the need for wave vector boosting [19]. Such boosting is required for SPR and guided mode resonance (GMR) excitation, through either prism or periodic grating which are not always convenient in practical applications. TPP is also polarization independent. Unlike SPR that only supports transverse magnetic (TM) wave, TPP can support both transverse electric (TE) and TM waves [18]. This allows the usage of unpolarized light sources in signal interrogation. TPP resonance can be interrogated by reflection, transmission, absorption spectroscopy, intensity interrogation, or phase measurement [20]. Due to its prominent characteristics, TPP has wide applications in sensing [21], lasing [22,23], perfect absorber [24], solar cell [25], optical filtering [26], and nonlinear optics [27]. In particular, TPP sensing has been demonstrated for detection of temperature [28], pressure [29], surface deformation [30], refractive index (R.I.) [31], organic vapor [32], greenhouse gases [33], liquids [34], fat concentration in milk [35], blood components [36], and theoretically predicted to be suitable for biosensing [37]. On the other hand, porous silicon (PSi) is an outstanding material for biosensing due to its high surface area of more than 100 m^2^ per 1 cm^2^ of planar surface area of etched silicon [38,39], versatile biofunctionalization chemistry, and CMOS (Complementary Metal Oxide Semiconductor) compatible fabrication process [40]. Researchers have proposed TPP sensors based on porous silicon DBR [41,42] and porous silicon resonant microcavity [33]. TPP based on 1D photonic crystal consisting of porous silicon membrane has also been demonstrated for nonspecific detection of organic solvents [43].

In this work, we demonstrate a TPP device based on porous silicon DBR as a biosensing platform for the detection of viral proteins—the N-protein of SARS-CoV-2. A TPP biosensor can be constructed by depositing noble metal thin film on porous silicon based DBR [44]. Incident white light can excite TPP resonance which has its resonant field energy confined near the interface between metal and DBR. The field strength decays exponentially further away from the interface into both the metal and the DBR. Further, due to the porous structure of porous silicon, a nanocomposite layer wherein metal nanoparticles are randomly distributed, can form in the top porous silicon layer of the porous silicon DBR [44]. As a result, the biomolecules infiltrate into the top porous silicon layer and interact with the strongest electrical field. This guarantees the highest sensitivity of detection. Another advantage of a porous silicon-based TPP biosensor is that, as will be demonstrated later, due to fast decaying profile of field strength inside and beyond thin metal film, refractive index (RI) changes beyond the first porous silicon layer of DBR has negligible effect on the TPP mode [45]. Thus, TPP is not sensitive to R.I. changes around the surface of the thin metal film. As will be demonstrated later in an experiment, this is advantageous in that it dispenses the rinsing procedure often required of biosensors to remove non-specific species adsorbed on biosensor surface.

The structural optimization of the TPP device has been carried out both theoretically, based on temporal coupled mode theory [44], and experimentally, based on optical characterization of fabricated TPP devices [46]. The goal is to achieve highest coupling efficiency of incident light into TPP mode, and the best possible field confinement. Adopting these results from the previous works, we design and optimize the structure of the porous silicon-based TPP biosensor. Then we use the optimized TPP biosensor to demonstrate nonspecific detection of RI liquids and sensitive and specific detection of SARS-CoV-2 N-protein.

## 2. Materials and Methods

Porous silicon DBR was fabricated by electrochemical anodization of 6-inch single crystalline silicon wafer (Boron doped p-type, 0.01 Ω∙cm resistivity, <100> crystal orientation) in 15% aqueous ethanoic hydrofluoric acid (HF) consisting of 3:7 *v*/*v* 50% aqueous HF and >98% ethanol. The designed central wavelength of the DBR bandgap is at 650 nm.

**Figure 1:**
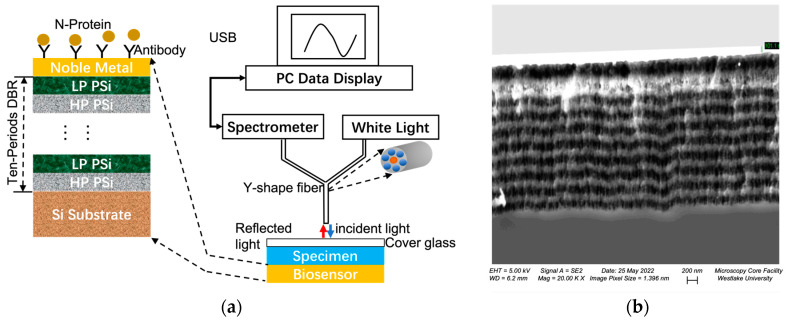
(**a**) Structural diagram and measurement setup of the TPP biosensor. The biosensor is a ten-period porous silicon DBR coated with Au thin film on top. The measurement system is reflection spectroscopy where a Y-shape fiber is used to provide incident white light and collect reflected light. (**b**) cross-sectional SEM image of the fabricated TPP biosensor. The layer-by-layer porous silicon structure comprising ten-period DBR is clearly visible, and the Au layer is the bright region on top of DBR.

The electrochemical etch conditions and the resulting porous silicon optical parameters, including the thickness and the effective refractive index of porous silicon based on Bruggeman effective medium theory [47], are listed in Table 1. The DBR has the structural configuration of [(LP PSi)/(HP PSi)]^10^, as shown in Figure 1a.

Afterwards, porous silicon was thermally oxidized in ambient air under 800 °C for 30 min to make the porous silicon stable and hydrophilic. Then, 5 nm Ti and 30 nm Au was consecutively deposited on the porous silicon DBR through magnetic controlled sputtering. Ti serves as adhesion layer between porous silicon and Au. The thickness of Au was optimized according to temporal coupled mode theory [44] and previous experimental results [46]. Finally, the 6-inch wafer was diced into 7 mm × 7 mm chip by dice saw equipment. Each chip serves as a TPP biosensor device.

The measurement of the biosensor is by reflection spectroscopy. As shown in Figure 1a, a white light source provides incident light which is guided by six circumferential fibers in a Y-shape fiber to be incident on the TPP biosensor surface. Light reflected from the TPP biosensor surface is collected by the central fiber of the Y-shape fiber and guided to a spectrometer for spectrum analysis.

For non-specific detection, liquids of various RIs consisting of 5% sucrose in deionized water (RI = 1.3648) and its serial dilutions are used as detection targets. The operation of RI sensing is as follows. First, the liquidous specimen is dropped on the biosensor surface, then a cover glass is placed on top of the specimen to flatten the liquid surface and facilitate spectral measurement as shown in Figure 1a. After the cover glass is placed, the reflection spectrum is collected. Then, the biosensor is rinsed with DI water and dried with nitrogen gas. Next, the liquid specimen of different RIs is applied on the biosensor surface, the cover glass placed on top, and reflection spectrum taken.

For specific detection of N-protein, the biosensors need functionalization with specific antibodies. The site where the antibody binds to the antigen is the Fab segment. ProteinA protein-modified TPP biosensor chips can be used to immobilize the antibody in a targeted manner, ensuring that the antibody binding site fixed on the chip faces upwards, reducing spatial hindrance, and improving the efficiency of N-protein antigen binding. The antibodies are immobilized on the TPP biosensor surface by the procedure shown in Table 2. Some of the challenges involved are as follows. The binding sites of antibodies to antigens is the Fab segment. The carboxyl chip, antibody hinge region, Fab, and Fc segments of the primary amino group can all be coupled with the carboxyl group, and the antibody binding sites may not necessarily face upwards, resulting in a decrease in subsequent antigen binding efficiency. ProteinA protein-modified chips can be used to selectively immobilize antibodies, ensuring that the fixed antibody binding site on the chip faces upwards, reducing steric hindrance, and improving the efficiency of N-protein antigen binding. Therefore, the ProteinA chip has better performance, and the main problems that might exist are sensitivity and non-specificity issues. Factors including the coating solution system (salt ion concentration ensuring protein activity, whether surfactants are added, etc.), antibody concentration (low concentration meaning less bound proteins and low biosensor signal value, while high concentration meaning denser coverage and possible steric hindrance which affect binding with subsequent antigen proteins), antibody isoelectric points (related to system pH), and antibody active sites, etc., need to be considered to optimize the sensitivity and specificity of the TPP biosensor.

The working mechanism of the TPP biosensor is based on perturbation theory [48], wherein biomolecules interact with confined electrical field of electromagnetic resonant mode of photonic spectrum. TPP is a resonant surface electromagnetic state with strong field confinement near the interface between metal and dielectric DBR. Any biomolecular binding will interact with the electrical field of the resonant mode and disturb its electrical field distribution profile. This perturbation results in a shift of the TPP resonant wavelength and reflection intensity in TPP reflection spectrum. The shift in wavelength is towards longer wavelength which is also called “redshift”. In this work, biomolecules are detected by detecting the redshift which is based on capturing the TPP resonant wavelength of both before and after biomolecular binding and calculating their difference. This has the advantage that any fluctuation of light intensity due to light source instability or ambient lighting change does not affect the detection of resonant wavelength, and thereafter the detection results. The operational procedure of biosensing is as follows. First, clinical specimens need to be taken from patients, such as saliva or swab specimen. Then the specimen is immediately placed in lysis buffer (viral transport medium (VTM) supplied by YOCON Biology Technology Company (Beijing, China)) to break down the virus and release its N-protein. This lysis sample containing N-protein can be used for biosensing directly since lysis solutions have been reported to keep the protein antigens bioactive for antibody recognition [49]. Alternatively, for biosensor characterization purposes, we can use commercially available N-proteins (Xlement Cat. No. C10002) as a target of detection and use different titration in PBS buffer to detect with the TPP biosensor. In this work, we use the latter approach for sensitivity, and limit the detection characterization and the former approach for specificity characterization. A volume of around 20 µL of the specimen is taken from the sampling tube and dropped onto the biosensor surface, then a cover glass is placed on top of the specimen to flatten the liquid surface and facilitate spectral measurement. After the cover glass is placed, the first reflection spectrum is collected as the spectrum before biomolecular binding. After binding reaction between N-proteins and antibodies completes which is about 10 min, the second reflection spectrum is collected as the spectrum after biomolecular binding. The two spectra of before and after reactions are compared and analyzed to determine the detection result. Note that rinsing the biosensor surface after binding reaction completes is not necessary, since any non-specific adsorption of bio species on thin metal film or even inside nanopores of thin metal film has little effect on the TPP resonance. This is because the electrical field in the thin metal film and beyond the thin metal film in the specimen reaction region is negligible. For a real time detection of N-protein binding with antibodies, the reflection spectrum of the TPP biosensor is automatically collected every 10 s in a ten-minute reaction process, without any disturbance of the biosensor or the cover glass.

For theoretical analysis, Transfer Matrix Method (TMM) [50] is used to simulate reflection spectrum of the TPP biosensor. For non-specific R.I. sensing, the liquids diffuse into the nanopores of gold thin film and porous silicon, causes changes in effective refractive index of porous silicon, and thus shifts the TPP characteristic resonant valley to longer wavelength (redshift). Liquids of different R.I. show different TPP resonant wavelengths. For specific biosensing, N-proteins bind with antibodies, which have been immobilized on biosensor beforehand, and causes changes in effective refractive index of porous silicon, and thus shifts TPP characteristic resonant valley. Different N-protein concentrations correspond to different amounts of N-protein binding with antibodies, causing different shift of TPP resonant valley. In all cases, more changes in effective R.I. of porous silicon generates more spectral shift.

The simulation of electrical field distribution inside the TPP device was carried out in COMSOL version 6.1. The resonance shift of the biosensor due to filling of the nanopores of porous silicon by liquid of various refractive indices was calculated in a MATLAB program based on TMM. The refractive index of porous silicon was calculated based on Bruggeman effective medium theory, with refractive index of nanopores being that of the liquid instead of air after pore filling by liquid. For the simulation of biosensing, we model the first porous silicon layer as a nanocomposite layer with 30% filling of nanopores with Au nanoparticles [44]. To avoid steric effects, the antibody immobilization is estimated to be 50% maximum coverage of pore surface [13,15]. The effective refractive index of porous silicon nanocomposite layer is also obtained by Bruggeman effective medium theory. Different amounts of N-protein binding with immobilized antibodies cause different amounts of redshift. Both antibodies and N-proteins are estimated to have refractive index of 1.45 [13,15].

## 3. Results and Discussion

**Figure 2:**
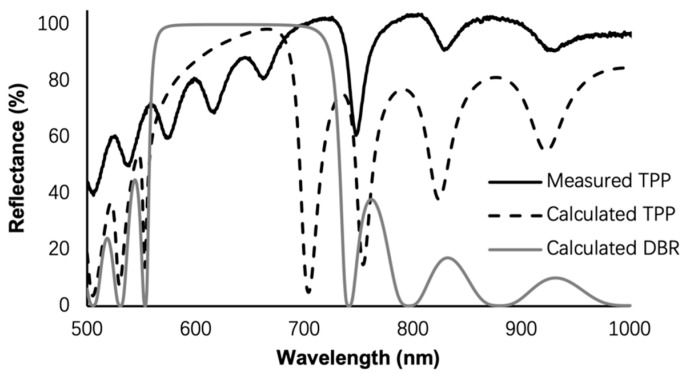
TMM calculated reflection spectra of the DBR and TPP, and measured reflection spectrum of the fabricated porous silicon TPP. Theoretical TPP resonance wavelength is inside the DBR bandgap and close to the band edge on the right. Fabricated device shows clear TPP resonance, with longer resonance wavelength than calculated. The discrepancies between measurement and TMM calculation are due to nonidealities of porous silicon and Au thin film thicknesses, and the fact that scattering and absorption losses of porous silicon are not modeled in TMM calculation.

**Figure 3:**
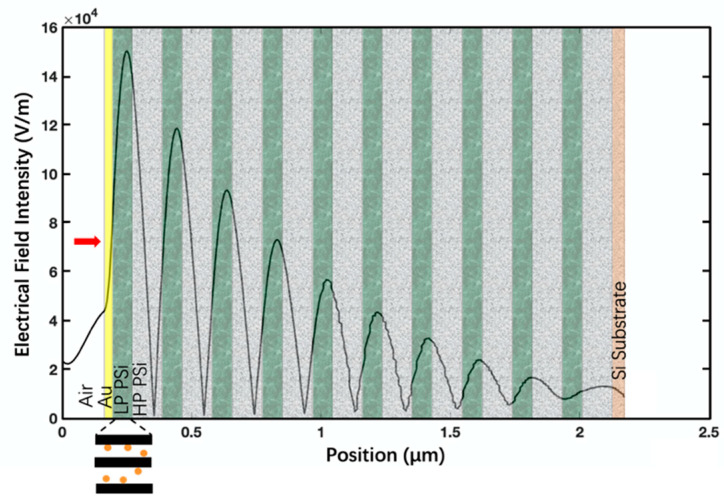
COMSOL simulated distribution of electrical field intensity of TPP biosensor based on ten-period porous silicon DBR. The polarization is transverse electric (TE), with red arrow at the left side indicating light of 1 W/m incident vertically from air. The highest peak of electrical field intensity resides in the first LP PSi layer. Au nanoparticles infiltrate into the first LP PSi layer to form a nanocomposite layer, which is displayed in the cartoon on the lower left of the figure.

Figure 1b shows the cross-sectional SEM image of the fabricated TPP device. The porous silicon DBR has 10 periods, and the 30 nm gold thin film is deposited on top with 5 nm Ti thin film as the adhesion layer between gold and porous silicon. The periodic structure of the fabricated porous silicon DBR is clearly visible. Figure 2 shows the calculated reflection spectra of the TPP biosensor and DBR reflector, and the measured reflection spectrum of the TPP biosensor. The calculation is based on the Transfer Matrix Method (TMM). We can see a salient resonant feature in spectrum of fabricated TPP which is due to the coupling of light energy into TPP resonance at the resonant wavelength. Figure 3 shows the electric filed distribution pattern of the TPP biosensor simulated by COMSOL. The field strength reaches its highest peak level inside the first porous silicon layer (the first LP PSi, as well as the nanocomposite layer, will be discussed later) and decays in both DBR and gold thin film.

Due to the porous nature of porous silicon, the conformally deposited Au thin film is also porous and the first LP PSi layer has Au nanoparticles infiltrating into nanopores. Thus, a nanocomposite layer forms with estimated filling ratio of 30% [44]. The nanocomposite is available for liquid or analyte infiltration. Any liquid infiltration or biomolecular binding inside the nanocomposite layer will cause an increase in refractive index and a perturbation of the electrical field.

**Figure 4:**
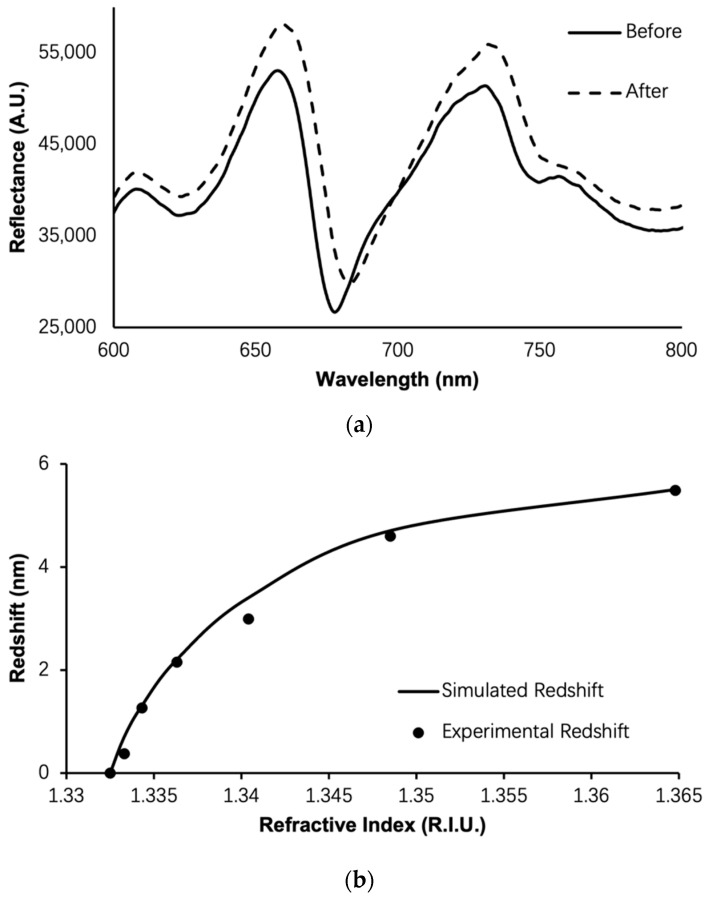
(**a**) Example of a redshift of the TPP resonance as a response to the perturbation of refractive index in the nanocomposite layer; (**b**) simulated (solid curve) and experimental (black circle) response curve of the TPP biosensor as liquids of varying refractive indices are applied on the biosensor surface.

This is the advantage of the PSi TPP biosensor since the analyte can interact with the peak electrical field and result in high sensitivity of detection. Figure 4a shows an example of TPP biosensor resonance redshift due to analyte binding in the nanocomposite layer. Figure 4b shows the simulated and experimental detection of liquids of varying R.I. Theoretical analysis agrees with experiment very well.

We also carried out detection of nucleocapsid proteins (N-protein) of SARS-CoV-2 with the TPP biosensor. As a key structural protein of SARS-CoV-2 with good immunogenicity, the N-protein is a good target to detect for COVID-19 diagnosis [51]. To guarantee specificity in biosensing, the TPP biosensor is first biofunctionalized with specific antibodies (see Table 2). Afterwards, varying concentrations of N-proteins were detected by the TPP biosensor.

**Figure 5:**
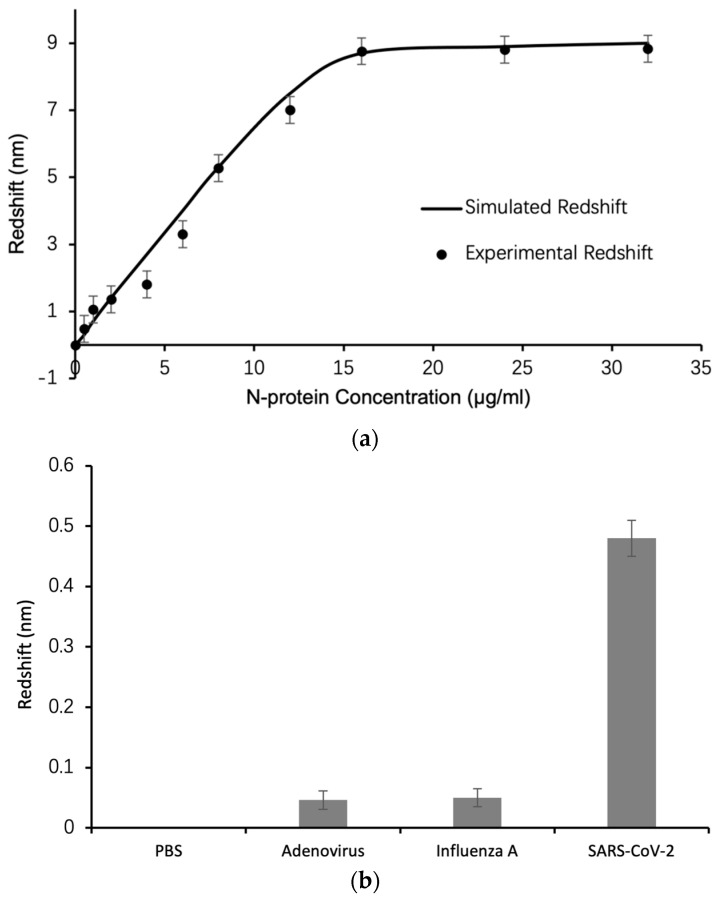

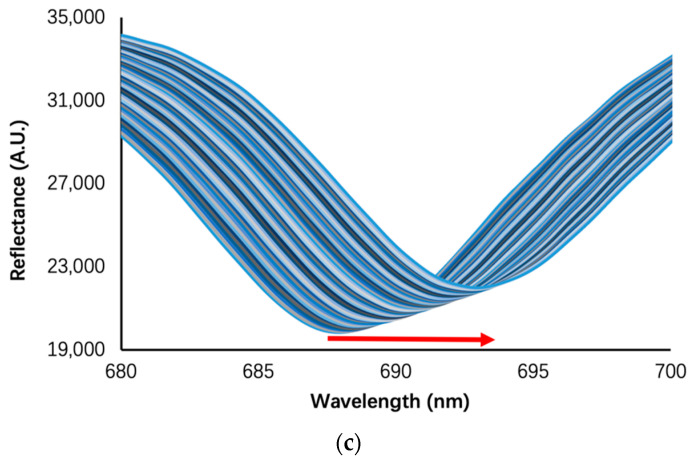
(**a**) Experimental (circle) and TMM calculated (solid curve) response of the TPP biosensor, that is, the amount of redshift of TPP resonance as function of N-protein concentrations with error bars showing standard error from five experiments; (**b**) comparison of TPP biosensor redshift for detecting clinical swab specimens containing SARS-CoV-2 virus, Influenza A virus and Adenovirus respectively, and PBS buffer only, with error bars showing standard error from five experiments; (**c**) real-time response of TPP biosensor for detection of 8 µg/mL of N-protein, in which the reflection spectrum of TPP biosensor is collected every ten seconds in a ten-minute detection process and the total redshift at the end (the red arrow) is up to 5.2 nm.

Figure 5a shows the response of the biosensor, which is the redshift of the TPP resonance, as a function of the N-protein concentration. The dots represent experimental data. The curve represents simulation through the Transfer Matrix Method (TMM) with calculations carried out in MATLAB version R2022b. The top porous silicon layer of the DBR is modeled as a nanocomposite consisting of porous silicon matrix and gold nanoparticles distributed randomly in the matrix with estimated 30% filling ratio. The antibodies as well as the N-proteins can infiltrate into this nanocomposite layer and interact with the strong electric field confined within the nanocomposite, resulting in high sensitivity biosensing. From Figure 5a, it can be seen that the experimental data match TMM modeling very well, which demonstrates that we have obtained a good structural modeling of the nanocomposite as well as the porous silicon DBR. Figure 5b demonstrates the specificity of the TPP biosensor by comparing the level of response signal (the amount of redshift) for clinical swab specimens taken from patients diagnosed as SARS-CoV-2 positive, Influenza A positive, and Adenovirus positive by RT-PCR technique. Also included for comparison is the PBS buffer. The response signal of the N-protein antibody biofunctionalized TPP biosensor for SARS-CoV-2 positive specimen is 10 times that of Influenza A and Adenovirus positive specimen. This demonstrates the good specificity of the biosensor towards N-protein detection and COVID-19 diagnosis.

From the experimental data, the highest sensitivity of the biosensor in the low target concentration region is 1.5 nm/(µg/mL). Given that the spectrometer can resolve minimum wavelength shifts of 0.01 nm, the limit of detection of the biosensor is 7 ng/mL. On the other hand, Figure 5c shows real-time detection of N-protein in a ten-minute scanning process, in which the reflection spectrum of the TPP biosensor is collected every 10 s. The TPP resonance wavelength shifts to longer wavelengths continuously as the N-protein is binding with specific N-protein antibodies. This functionality of the biosensor can serve as a real-time detection tool for the analysis of dynamics of biomolecular interactions.

Table 3 shows a comparison of state-of-the-art bioanalytical technologies with the TPP biosensor proposed in this work for the purpose of COVID-19 diagnosis. The RT-PCR technique has high sensitivity, good specificity, and high throughput, but requires a clean environment, bulky equipment, and well-trained personnel. It has a turnaround time of at least 4 h, making it unsuitable for rapid onsite detection or large-scale population screening. To complement RT-PCR, antigen and antibody detection based on lateral flow technology has been used in clinical diagnostics. They are on-site deployable with simple operation, have no need for equipment, and have a fast turnaround of about 10–15 min. However, they have their own limitations. The sensitivity of antigen detection is not satisfactory, making the detection result accurate only in the first week of disease onset. For antibody detection, the target of detection, which are typically IgG and IgM antibodies, can be related with other infections such as common cold or flu. Therefore, positive results of antibody tests may not necessarily point to COVID-19 and further diagnosis by RT-PCR or computed tomography (CT) is required to accurately diagnose. Furthermore, neither antigen test nor antibody test has high throughput since they are designed mainly for home test or self-test. For biosensors proposed in this work, high sensitivity, good specificity, and high throughput [14] can all be obtained. It requires little training, has fast turnaround time of 10 min, and can be deployed in the field. The only disadvantage is that it requires equipment to carry out detection, either handheld for home use or self-rest, or high throughput for benchtop analytical lab application. However, compared with RT-PCR equipment, the biosensor detection equipment is much simpler and easier to operate, has no requirement on cleanness of environment, and thus is much more suitable for rapid onsite detection. In summary, Table 3 demonstrates the advantages of the proposed TPP biosensor for rapid onsite detection with good sensitivity, specificity, and high throughput capability. The TPP biosensing platform combines the advantages of the two mainstream COVID-19 detection technologies currently in clinical use—RT-PCR and lateral flow detection.

To improve the performance of TPP biosensors, there are two major directions of research. The first is to explore other plasmonic materials, such as graphene to replace Au thin film. Graphene is a two-dimensional nanomaterial with tunable conductivity. Therefore, it allows the tuning of the TPP resonance wavelength and the field confinement capability. The second is to design aperiodic DBR instead of periodic structure for TPP device. Aperiodic DBR for constructing TPP has the advantage of more flexible control of photonic bandgap and reflection spectrum, thus enabling the custom design of reflection spectral characteristics such as the quality factor of TPP resonance and field confinement profile. All the research should aim at enhancing the sensitivity of the TPP biosensor.

## 4. Conclusions

We have proposed a porous silicon-based TPP biosensor. We have optimized the structure of TPP biosensor and analyzed the performance in terms of nonspecific detection of R.I. liquids and specific detection of N-protein of SARS-CoV-2. Theoretical calculations based on the Transfer Matrix Method were compared with experimental results, and good agreements were obtained. From COMSOL simulation, it is found that the TPP biosensor has strong electrical field confinement in the vicinity of the interface between porous silicon and thin metal film. Specifically, the strongest peak of electrical field resides in the first porous silicon layer of the porous silicon DBR. This layer is available for Au nanoparticle infiltration which forms the porous silicon nanocomposite layer. More importantly, the nanocomposite layer is also available for liquid filling, biomolecular infiltration, and biomolecular binding. Both R.I. sensing and N-protein detection via the sensitive TPP biosensor have been demonstrated. The sensitivity of the biosensor can be up to 1.5 nm/(µg/mL), and the limit of detection can reach down to 7 ng/mL. High sensitivity of the TPP biosensor originates from strong interaction between biomolecules and electrical field confined in the nanocomposite layer. The TPP biosensor has the advantages of simplified measurement through reflection spectroscopy without any requirement on polarization selection or wave vector boosting, no need for rinsing of biosensor surface after antigen-antibody reaction, real time monitoring of biomolecular binding process, and CMOS compatible fabrication process. In the future, the sensitivity of the TPP biosensor can be further enhanced by employing sandwich assay which can amplify the level of response significantly [55]. To lower the limit of detection, an advanced spectrometer of 1 pm resolution in wavelength shift can be used. Moreover, simultaneous detection of multiple analytical targets, such as antigens, antibodies, and nucleic acids, with a TPP biosensor array can be realized to capture the presence of pathogens in specimens more efficiently. TPP biosensing of oligo nucleotides and specific DNA sequences can lead to differentiation between various strains of SARS-CoV-2 and viral mutations. Finally, to put TPP biosensing platform into practical applications, especially in resource limited settings, mass production of TPP biosensors (preferably through CMOS compatible process since every step of TPP device fabrication including porous silicon etching is CMOS compatible and CMOS infrastructure is relatively mature in semiconductor industry), has to be realized so that the material and process cost per chip can be dramatically lowered through highly efficient volume production. It will be a commercially viable solution for in vitro diagnostics market if its price is comparable with the now commercially available antigen test kit. In summary, porous silicon-based TPP biosensor is an outstanding technological platform for point-of-care test in many areas such as biomedical diagnostics, food safety, biosafety, environment protection, and epidemic control.

## Figures and Tables

**Table 1:** Porous silicon etch conditions and optical parameters.

Structure	Porosity	Current Density	Etch Time	Bruggeman Effective R.I.	Thickness
LP PSi	52%	5 mA/cm^2^	20 s	2.08	100 nm
HP PSi	76%	48 mA/cm^2^	6 s	1.41	150 nm

LP: Low Porosity; HP: High Porosity.

**Table 2:** Procedure for immobilization of specific N-protein antibodies on TPP biosensor.

No.	Step Name	Step Operation
1	Chip cleaning	Clean the biosensor chips using a plasma cleaning machine, ethanol, isopropanol, and ultrapure water, and then place them in a 96 well plate container for later use.
2	Sulfhydryl proteinA modified chip	Dilute mercapto proteinA (Xlement Cat. No. G60001) with ultrapure water to a working concentration of 50 μg/mL, take an appropriate amount of mercapto proteinA solution and add it to the biosensor chip surface. Leave at 4 °C overnight or 37 °C for 2 h.
3	Preparation of coating antibody solution	Take COVID-19 N-protein antibody (Xlement Cat. No. C10002), use coupling buffer solution (Xlement Cat. No. S20029) to prepare 50 μg/mL of coating antibody solution.
4	Chip directed immobilization of antibodies	Take an appropriate amount of 50 μg/mL of coating antibody solution and apply on the surface of the biosensor chip and react at 37 °C (with shaking) for 20–30 min. After reaction completes, clean the biosensor chip twice with Phosphate-buffered saline (PBS) buffer solution (pH~7.4).
5	Closure	Take an appropriate amount of sealing solution (Xlement Cat. No. G30004) and add it to the surface of the biosensor chip. Leave it at 37 °C for 30 min. After removing and drying the sealing solution, the chips can be used directly for bioanalytical detection assay. Alternatively, perform the following steps before storing chips for future use.
6	Protection	Take an appropriate amount of protective solution (Xlement Cat. No. G30006) and add it to the surface of the biosensor chip. Place it at 37 °C for 30 min, and then remove and dry the protective solution.
7	Chip drying	Place the modified chip in a 37 °C oven and dry for 5 min.
8	Plastic sealing	Use a sealing machine to vacuum seal the wrapped chips and refrigerate them for storage.
9	Storage	The sealed chip is generally stable with shelf life of 3 days at 37 °C and 6 months at 4 °C. Store the vacuum sealed chips in dry and dark conditions can also extend the shelf life.
10	Biosensing	Open the chip sealing. Drop 20 µL of N-protein solution (Xlement Cat. No. C10002) in varying concentrations in PBS buffer on biosensor surface. Put on cover glass and take spectral measurement with fiber spectrometer.

**Table 3:** A comparison of the TPP biosensor technology proposed in this work with state-of-the-art COVID-19 diagnostic techniques reported in the literature.

Technologies	Target	Sensitivity	Specificity	Advantages	Disadvantages
Reverse transcription polymerase chain reaction (RT-PCR) [52]	Specific gene sequence, such as ORF1ab	>90%	Nearly 100%	Accurate result, current gold standard, high throughput.	Need clean environment, complex equipment, and staff training, slow turnaround
Antigen detection by lateral flow [53]	Viral proteins such as N-protein Or S-protein	37.7–99.2%	92.4–100%	Rapid and onsite detection, no need for equipment.	Results not accurate due to dynamic antigen secretion and sensitivity limitations.
Antibody detection by lateral flow [54]	Immune globulin such as IgG or IgM	41.1–95%	98.6–99.8%	Rapid and onsite detection, no need for equipment.	Positive results require further verification by RT-PCR or CT scan.
TPP biosensor (this work)	N-protein	>90%	>95%	Rapid and onsite detection, high throughput.	Need handheld or desktop equipment

## Data Availability

The data presented in this study are available on request from the corresponding author. The data are not publicly available due to privacy.
